# In Vitro Antioxidant Activity of Peptides from Simulated Gastro-Intestinal Digestion Products of *Cyprinus carpio haematopterus* Scale Gelatin

**DOI:** 10.3390/foods8120618

**Published:** 2019-11-25

**Authors:** Feng Xiao, Shengjun Chen, Laihao Li, Jialiang He, Weiwei Cheng, Guoyan Ren

**Affiliations:** 1 College of Food and Bioengineering, Henan University of Science and Technology, Luoyang 471023, China; xfeng@haust.edu.cn (F.X.); he-jliang@163.com (J.H.);; 2 Key Laboratory of Aquatic Product Processing, Ministry of Agriculture and Rural Affairs, National R&D Center for Aquatic Product Processing, South China Sea Fisheries Research Institute, CAFS, Guangzhou 510300, China; laihaoli@163.com

**Keywords:** antioxidant, simulated gastro-intestinal digestion, *Cyprinus carpio haematopterus* scale, gelatin

## Abstract

A two-stage simulated gastro-intestinal (GI) digestion model (2 h pepsin treatment and subsequent 2 h pancreatin treatment at 37 °C) was used to explore the antioxidant activity of the digested products of *Cyprinus carpio haematopterus* scale gelatin with different molecular weights (MW). From the gastric phase to the intestinal phase, the hydrolysis degree of the products increased from 2.6 ± 0.4% to 16.9 ± 0.7%. The fraction of 0–1 kDa (JCP3) exhibited the best antioxidant activities in hydroxyl radical scavenging, reducing power, and metal chelating activity. The fraction of 1–3 kDa (JCP2) exhibited the best 1,1-diphenyl-2-picrylhydrazyl (DPPH) radical scavenging activity. However, the fractions of 0–1 kDa (JCP3) and 1–3 kDa (JCP2) showed similar inhibitory activity of lipid peroxidation. The results indicated that *Cyprinus carpio haematopterus* scale gelatin can be digested in the gastrointestinal tract. Furthermore, the digested products had antioxidant activity.

## 1. Introduction

Gelatins, the heat denaturation products of collagens have been widely used as food process aids and additives in the food industry. Type-I collagens are mainly from bovine or porcine dermis [1]. However, they have also been extracted from skin, bones, and scales of freshwater and marine fish [2,3,4,5,6]. Therefore, collagens from aquatic animals have a broad application prospect.

Recently, peptides with potential antioxidant activity have been obtained from various materials, such as soy proteins [7,8], caseins [9,10], egg proteins [11], aquatic product proteins [12,13,14], and other materials.

The presence of a certain amount of free radicals is advantageous to cells. However, excessive free radicals or weakened antioxidant defense systems can cause cell damage [15]. Enzymatic systems and chemical scavengers can remove free radicals formed in cells. The free radicals are involved in the development of various pathological processes [16]. Due to the negative effects of the oxidative processes, the inhibition of oxygen free radicals inside organisms is of great importance. Many food compositions, such as polyphenols, ascorbic acids, and protein hydrolysates, show the antioxidant activity.

The biologically active peptides in proteins can be obtained during gastrointestinal digestion. Studies show that biological properties of protein hydrolysates depend on the molecular weight [7,8,17]. At present, the application of collagens in functional foods and cosmetics has become a research hotspot. Normally, researchers try to hydrolyze collagen or gelatin with various enzymes to obtain active peptides. However, these peptides are likely to be further hydrolyzed after entering the digestive tract, thus losing their activity. Simulated digestion can largely reflect the function of collagen or gelatin as a food component after ingestion by the human body. As a connective tissue protein, the intact protein cannot be absorbed. However, the digestive characteristics of collagen or the biological activities of collagen digested products have been seldom reported. Collagen from different sources has different hydrolytic properties, and the composition and function of hydrolysates are also different. In addition, collagen in processed food will exist in the form of gelatin. So far, there is no report on antioxidant activity of peptides from simulated gastro-intestinal digested products of *Cyprinus carpio haematopterus* scale gelatin. This study aimed to explore the feasibility of the application of *Cyprinus carpio haematopterus* scale collagen in functional food. In the study, a simulated digestion process was performed on *Cyprinus carpio haematopterus* scale gelatin. The antioxidant activities of the digested products were detected to evaluate the potential biological activity of *Cyprinus carpio haematopterus* scale gelatin during the digestive process.

## 2. Materials and Methods

### 2.1. Materials

Live *Cyprinus carpio haematopterus* with the body weight of 1300 ± 50 g were bought from the market of Luoyang, China. Gelatins were extracted from fish scales in three sequential steps. Firstly, fish scales were demineralized according to the method of Feng Xiao et al. [6]. Then the non-collagenous proteins were removed with 0.2 M NaOH solution at 4 °C for 24 h. Finally, gelatins were isolated by hot water treatment at 80 °C for 2 h.

### 2.2. Chemicals and Reagents

Trolox, pepsin, pancreatin, 1,1-diphenyl-2-picrylhydrazyl (DPPH), and ferrozine were purchased from Sigma-Aldrich, Inc. (St. Louis, MO, USA). Other chemicals used in the experiments were of analytical grade.

### 2.3. In Vitro Simulated GI Digestion

A two-stage simulated gastro-intestinal (GI) digestion model (2 h pepsin treatment and subsequent 2 h pancreatin treatment at 37 °C) was used to hydrolyze the *Cyprinus carpio haematopterus* scale gelatin sample according to the method of Lijun You et al. [18]. The *Cyprinus carpio haematopterus* scale gelatin solution (2% *w*/*v*) was adjusted to pH 2.0 with 1.0 M HCl before pepsin (2%, *w*/*w*) hydrolysis. Then the solution was adjusted to pH 7.5 with 1.0 M NaOH and pancreatin (4%, *w*/*w*) was added.

To terminate the digestion, the samples were inactivated at 80 °C for 15 min. The digested products were centrifuged at 11,000× *g* for 15 min.

### 2.4. Measurement of Hydrolysis Degree (DH)

Firstly, the amount of free amino groups in the *Cyprinus carpio haematopterus* scale gelatin digested samples was estimated via the ninhydrin reaction [19]. Then the samples were hydrolyzed with 6 M HCl at 130 °C for 4 h. The amount of total amino groups was determined.

(1) $$DH=\frac{\mathrm{The}\;\mathrm{amount}\;\mathrm{of}\;\mathrm{free}\;\mathrm{amino}\;\mathrm{groups}}{\mathrm{The}\;\mathrm{amount}\;\mathrm{of}\;\mathrm{total}\;\mathrm{amino}\;\mathrm{groups}}\times 100$$

### 2.5. Measurement of Consituents of Cyprinus Carpio Haematopterus Scale

The protein content of *Cyprinus carpio haematopterus* scale was assayed according to the Kjeldahl method. The moisture content of *Cyprinus carpio haematopterus* scale was assayed according to the loss of water on drying under normal pressure at 105 °C. The mineral content of *Cyprinus carpio haematopterus* scale was assayed according to the method of burning at 550 °C. The collagen content of *Cyprinus carpio haematopterus* scale was assayed according to the method of Feng Xiao et al. [6]

### 2.6. Isolation of Peptide Fractions from GI Digestion

The digested products were subjected to sequential ultrafiltration (UF) steps at 4 °C. Three fractions of JCP1 (molecular weight >3 kDa), JCP2 (molecular weight 1–3 kDa), and JCP3 (molecular weight <1 kDa) were prepared. The protein content percentage of each fraction was assayed according to the Kjeldahl method. The free amino groups percentage of each fraction was determined according to ninhydrin colorimetry.

### 2.7. Determination of Antioxidant Activities

#### 2.7.1. Hydroxyl Radical Scavenging Activity Assay

The samples of JCP1, JCP2, and JCP3 were prepared with distilled water as solutions with concentrations from 0 to 2.0 mg protein/mL. Hydroxyl radical scavenging activity of *Cyprinus carpio haematopterus* scale gelatin digested products was assayed according to the method of Lijun You et al. [18]. The mixture included 600 ìL of 1,10-phenanthroline (5.0 mM), 600 ìL of ethylene diamine tetraacetic acid (15 mM), 600 ìL of FeSO_4_ (5.0 mM), and 400 ìL of sodium phosphate buffer (0.2 M, pH 7.4). Then the digested products of *Cyprinus carpio haematopterus* scale gelatin (600 ìL, 2.0 mg/mL) and H_2_O_2_ (800 ìL, 0.1%) were added. The absorbance was determined 60 min later, at a wavelength of 536 nm.

(2) $$\mathrm{Hydroxyl}\;\mathrm{radical}\;\mathrm{scavenging}\;\mathrm{activity}\;(\%)=\frac{A_{s}-A_{0}}{A_{c}-A_{0}}\times 100$$

where A_s_, A_0,_ and A_c_ are the absorbance of the test sample, the blank sample, and the control sample, respectively.

#### 2.7.2. DPPH Radical Scavenging Activity Assay

DPPH radical scavenging activity of the JCP1, JCP2, and JCP3 samples was determined according to the method of Jinle Xiang et al. [20,21]. DPPH radical scavenging activity of the *Cyprinus carpio haematopterus* scale gelatin digested product was calculated.

(3) $$\mathrm{DPPH}\;\mathrm{radical}\;\mathrm{scavenging}\;\mathrm{activity}\;(\%)\;=\;\frac{A_{\mathrm{DPPH}\;\mathrm{sample}}-A_{\mathrm{control}}\;}{A_{\mathrm{DPPH}\;\mathrm{blank}}}\times \;100$$

where A_DPPH sample_ is the absorbance of 2 mL of *Cyprinus carpio haematopterus* scale gelatin digested product mixed with DPPH solution, A_control_ is the absorbance for 2 mL of *Cyprinus carpio haematopterus* scale gelatin digested product mixed with 2 mL of 95% ethanol and A_DPPH blank_ is the absorbance for ethanol mixed with DPPH solution.

#### 2.7.3. Reducing Power Assay

The reducing power of the JCP1, JCP2, and JCP3 samples was determined according to the method of Lijun You et al. [18]. The absorbances at 700 nm were used to reflect the activity of *Cyprinus carpio haematopterus* scale gelatin digested samples.

#### 2.7.4. Metal Chelating Activity

Metal ion chelating activity was measured according to the method described by Xie et al. [22] with minor modification. The JCP1, JCP2, and JCP3 samples (1 mL) were premixed with FeCl_2_ solution (0.05 mL, 2 mM) and distilled water (1.85 mL). Afterwards, ferrozine solution (0.1 mL, 5 mM) was added. Ten minutes later the absorbance at 562 nm was determined. Distilled water was used as the blank solution.

(4) $${\mathrm{Fe}}^{2+}\;\mathrm{chelating}\;\mathrm{activity}\;(\%)=\frac{A_{0}-A_{S}}{A_{0}}\;\times \;100$$

where A_S_ and A_0_ are the absorbances of the test sample and the blank, respectively.

#### 2.7.5. Measurements of the Lipid Peroxidation Inhibition Activity

The activity of the JCP1, JCP2, and JCP3 samples in a linoleic acid emulsion system was measured according to the methods of Lijun You et al. [18]. The peroxide value was determined by monitoring the absorbance at 500 nm until the absorbance of the control reached the maximum value.

(5) $$\mathrm{The}\;\mathrm{lipid}\;\mathrm{peroxidation}\;\mathrm{inhibition}\;\mathrm{activity}\;(\%)=\frac{{1-(A}_{s,t=48h}{-A}_{s,t=0h})}{A_{0,t=48h}{\;-\;A}_{0,t=0h}}\;\times \;100$$

where A_s,t=48h_ and A_s,t=0h_ are the absorbances of the JCP1, JCP2, and JCP3 samples at 48 h and 0 h, respectively and A_0,t=48h_ and A_0,t=0h_ are the absorbances of the control at 48 h and 0 h, respectively.

## 3. Results and Discussion

### 3.1. The Basic Components of Cyprinus Carpio Haematopterus Scale

The basic components of *Cyprinus carpio haematopterus* scale are shown in Table 1. The results indicated that the protein content in *Cyprinus carpio haematopterus* scale was very high. Furthermore, the percent of collagen in *Cyprinus carpio haematopterus* scale was about 13.43 ± 0.46%. That means the collagen accounts for about 23% of total protein in *Cyprinus carpio haematopterus* scale. Therefore, the *Cyprinus carpio haematopterus* scale may be utilized as the source of collagen.

### 3.2. DH of GI Digests

**Figure 1:**
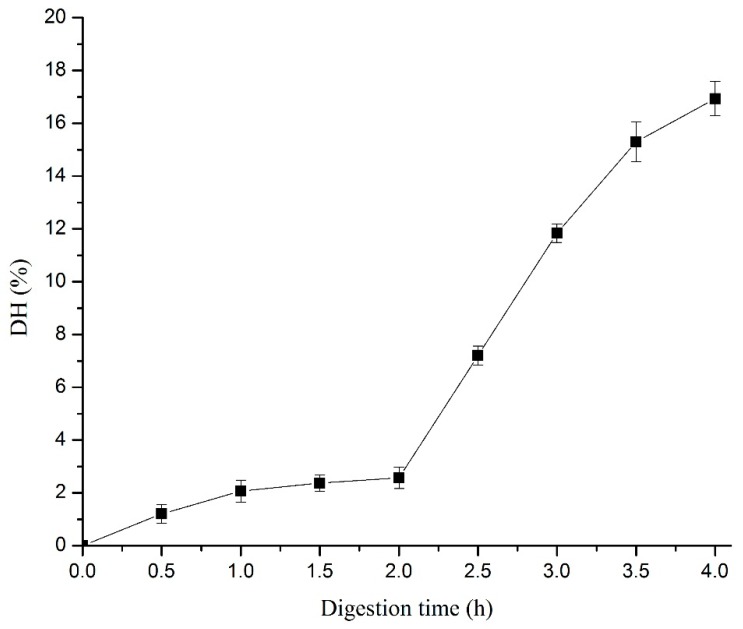
Changes in hydrolysis degree (DH) of *Cyprinus carpio haematopterus* scale gelatin following the simulated gastro-intestinal digestion process. The gelatin was digested with pepsin in the first 2.0 h, and then treated with pancreatin from 2.0 to 4.0 h.

A two-stage simulated gastro-intestinal (GI) digestion model (2 h pepsin treatment and subsequent 2 h pancreatin treatment at 37 °C) was used to hydrolyse the *Cyprinus carpio haematopterus* scale gelatin. As shown in Figure 1, the DH of *Cyprinus carpio haematopterus* scale gelatin increased slightly (*p* > 0.05) when the gelatin was digested by pepsin for 2 h. This suggested that peptide bonds are seldom broken during pepsin digestion. Studies have shown that pepsin can only enzymatically hydrolyze the cross-linked region of the collagen [23]. The results showed that pancreatin led to a significant increase in DH from 2.6 ± 0.4% to 16.9 ± 0.7% (*p* < 0.05). Therefore, pancreatin may hydrolyze the collagen into peptides and possibly amino acids.

### 3.3. Molecular Weight Distribution of GI Digestion

The digested products of *Cyprinus carpio haematopterus* scale gelatin were subjected to sequential ultrafiltration (UF) steps at 4 °C. Three fractions with different molecular weight of JCP1, JCP2, and JCP3 were obtained. The content of protein and percentage of free amino groups in different fractions of *Cyprinus carpio haematopterus* scale gelatin digested products are shown in Table 2. The protein contents in three fractions were 46.2%, 29.3%, and 23.5%, respectively. The percentage of free amino groups in three fractions were 16.5%, 19.3%, and 61.8%, respectively.

### 3.4. Antioxidant Activities of Cyprinus carpio haematopterus Scale Gelatin Digested Products

#### 3.4.1. Hydroxyl Radical Scavenging Activity

**Figure 2:**
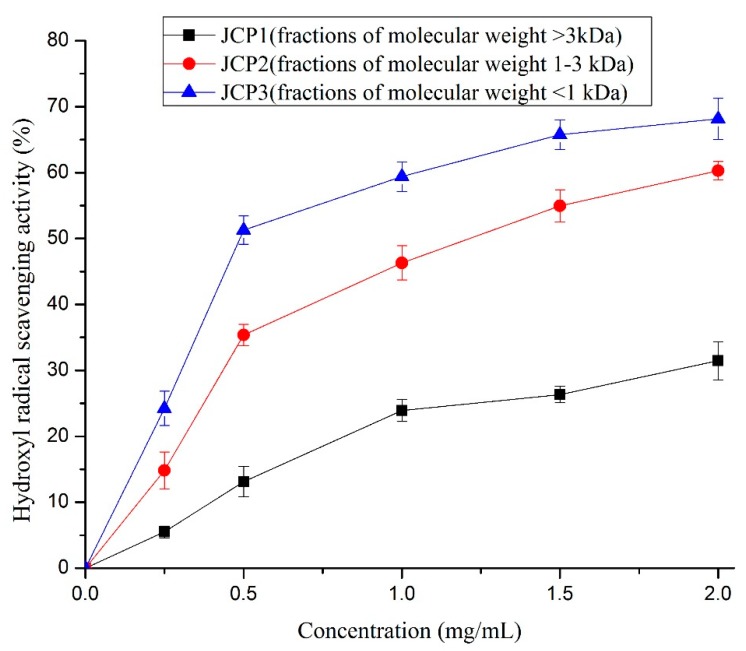
Hydroxyl radical scavenging activities of JCP1, JCP2, and JCP3. The JCP1, JCP2, and JCP3 are fractions of molecular weight >3 kDa, 1–3 kDa, and <1 kDa isolated *Cyprinus carpio haematopterus* scale gelatin digested products.

Studies have shown that hydroxyl radicals can induce severe cell damage because of their strong reactivity [22]. Accordingly, the scavenging of the hydroxyl radical is probably an effective defense mechanism of a living body [24]. The hydroxyl radical scavenging activity of *Cyprinus carpio haematopterus* scale gelatin digested products at different concentrations is shown in Figure 2. The hydroxyl radical scavenging activity of the digested products increased with the peptide concentration. It showed a clear linear relationship with the *Cyprinus carpio haematopterus* scale gelatin digested product concentration when the concentration was lower than 0.5 mg/mL. The two fractions with lower molecular weight (JCP2 and JCP3) had strong activity and the IC_50_ values of them were 1.14 mg/mL and 0.57 mg/mL, respectively.

#### 3.4.2. DPPH Radical Scavenging Activity

**Figure 3:**
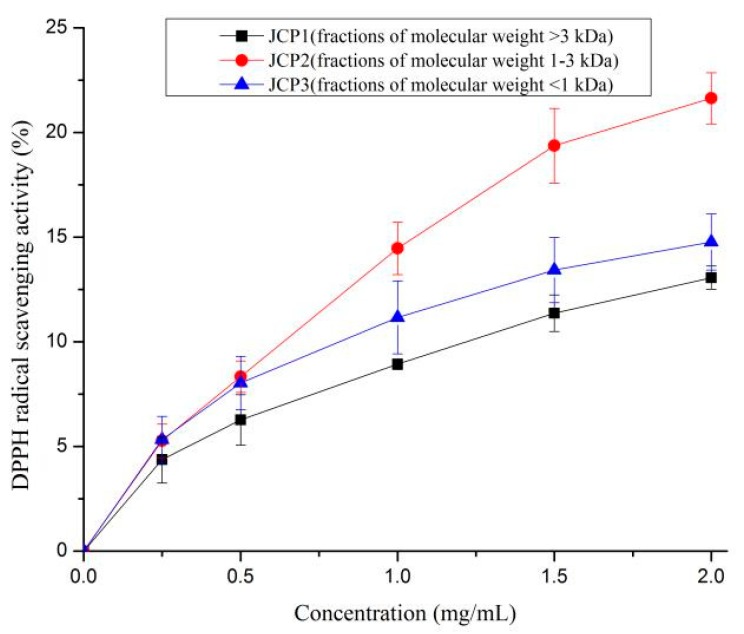
DPPH radical scavenging activities of JCP1, JCP2, and JCP3. The JCP1, JCP2, and JCP3 are fractions of molecular weight >3 kDa, 1–3 kDa, and <1 kDa isolated *Cyprinus carpio haematopterus* scale gelatin digested products.

DPPH radicals can loosen the chromophore when receiving an electron from any hydrogen donor. In general, the radical is usually employed to detect the antioxidant activity of constituents due to its stability and convenience in the isolation process [25]. When free radicals were scavenged, the absorbance of ethanolic DPPH solution at 517 nm decreased gradually and the color of the solution changed from purple to yellow. As shown in Figure 3, the activities of the three fractions increased with the peptide concentration. The fraction of JCP2 exhibited the highest activity compared to JCP1 and JCP3. The IC50 values of JCP2 and JCP3 were 4.46 mg/mL and 6.97 mg/mL, respectively.The results indicated that low molecular weight peptides of *Cyprinus carpio haematopterus* scale gelatin digested products were more effective DPPH radical scavengers than high molecular weight peptides. Wang et al. [26] have reported that low molecular weight peptides from the blue mussel with MW < 1 kDa possesse the higher activity. In contrast, it has also been reported that the fraction of >5 kDa from wheat gluten possesses the higher activity [27].

#### 3.4.3. Reducing Power

**Figure 4:**
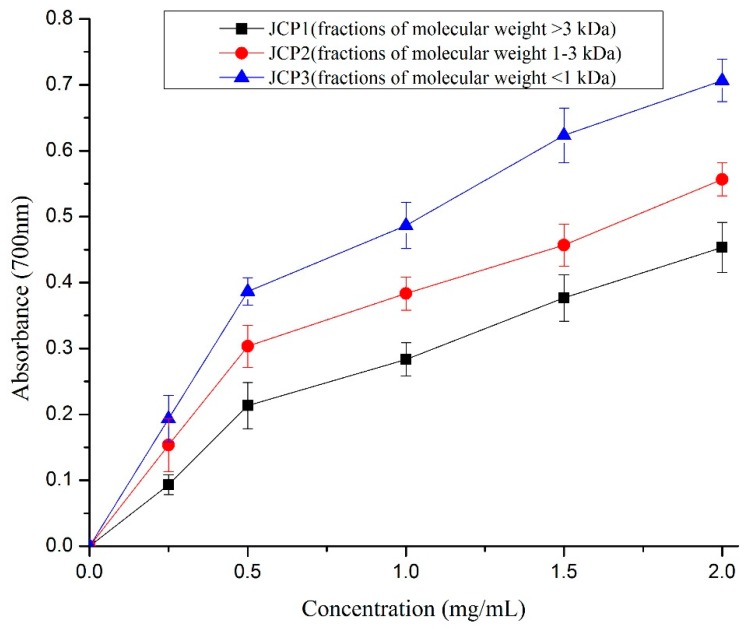
The reducing power of JCP1, JCP2, and JCP3. The JCP1, JCP2, and JCP3 are fractions of molecular weight >3 kDa, 1–3 kDa, and <1 kDa isolated *Cyprinus carpio haematopterus* scale gelatin digested products.

The reducing power is in direct proportion to the absorbance at 700 nm in this system. Some studies indicated that the antioxidant activities were directly correlated with reducing power activity of peptides [28]. The reducing power of *Cyprinus carpio haematopterus* scale gelatin digested products is shown in Figure 4. The results indicated that the peptide concentration is responsible for the reducing power of *Cyprinus carpio haematopterus* scale gelatin digested products. The fraction of JCP1 exhibited the highest reducing power, while the other two fractions, JCP2 and JCP3, with lower molecular weight exhibited lower activity. The results are in agreement with previous report that low molecular weight peptides of <1 kDa in some protein hydrolysates had the higher reducing power [29]. Furthermore, it was reported that the hydrophobic amino acids were responsible for the reducing power [30].

#### 3.4.4. Chelating Activity of Cu^2+^

**Figure 5:**
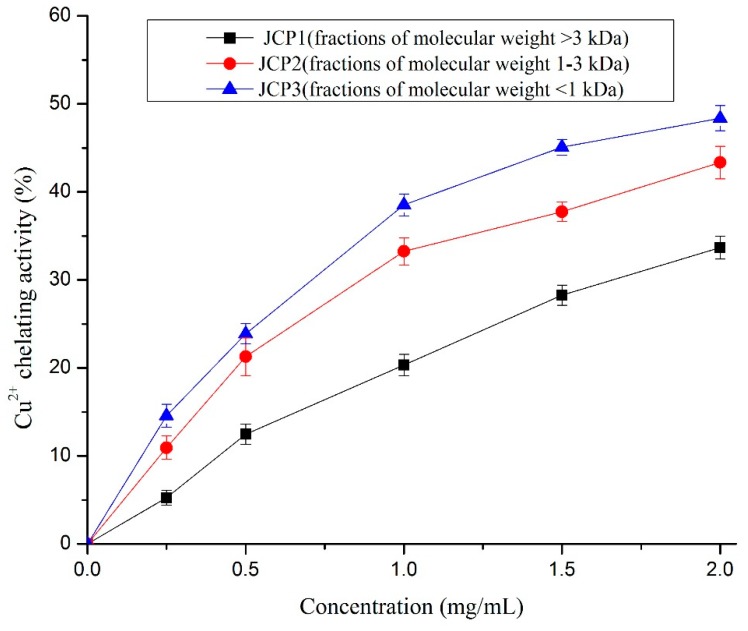
The chelating activities of JCP1, JCP2, and JCP3. The JCP1, JCP2, and JCP3 are fractions of molecular weight >3 kDa, 1–3 kDa, and <1 kDa isolated *Cyprinus carpio haematopterus* scale gelatin digested products.

Previous research suggested that the chelating of metal ions contributes to reducing the probability of the superoxide anion forming a hydroxyl radical [28]. Some compounds exhibit antioxidant activity and affect the peroxidation process via interfering with the catalytic activity of metal ions. [22]. As shown in Figure 5, the metal chelating activity of *Cyprinus carpio haematopterus* scale gelatin digested products increased with the increase of the peptide concentration used in the test. The fraction of JCP3 showed the strongest copper chelating ability (about 48.4 ± 1.4% at 2 mg/mL) compared with the fractions of JCP1 and JCP2. Similar results of silver carp protein hydrolysates were reported by Dong et al. [31].

#### 3.4.5. Lipid Peroxidation Inhibition Activity

**Figure 6:**
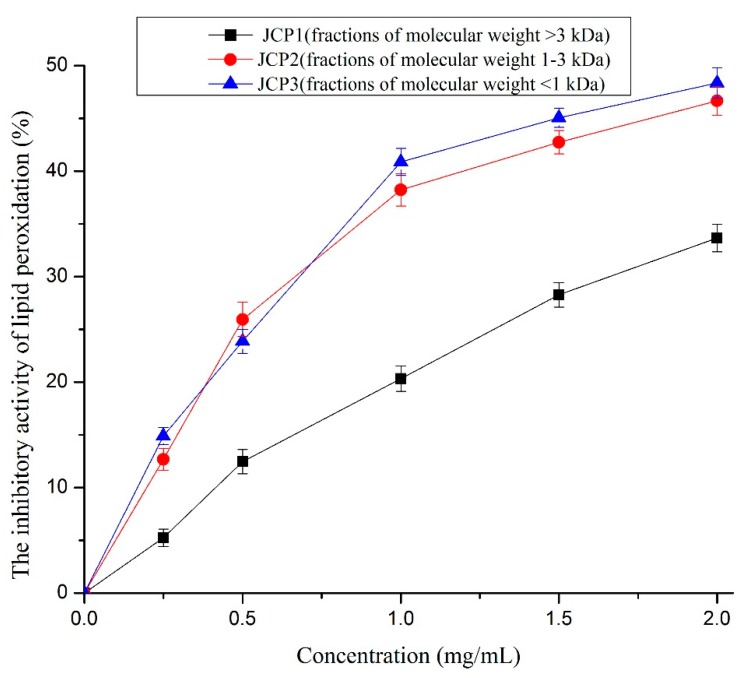
The inhibitory activities of JCP1, JCP2, and JCP3. The JCP1, JCP2, and JCP3 are fractions of molecular weight >3 kDa, 1–3 kDa, and <1 kDa isolated *Cyprinus carpio haematopterus* scale gelatin digested products.

The lipid peroxidation inhibition activity of *Cyprinus carpio haematopterus* scale gelatin digested products is shown in Figure 6. It can be seen that the fraction of 1–3 kDa (JCP2) and the fraction of <1 kDa (JCP3) in *Cyprinus carpio haematopterus* scale gelatin digested products offered substantial inhibition against the peroxidation of linoleic acid, whereas the fraction of >3 kDa (JCP1) offered less effective inhibition. The fraction of 1–3 kDa (JCP2) and the fraction of <1 kDa (JCP3) showed no significant difference in the inhibitory activity (*p* > 0.05) and the IC_50_ values of the two fractions were 2.31 mg/mL and 2.58 mg/mL, respectively. Therefore, the relative molecular mass of the peptide was not the decisive factor in the inhibitory activity of lipid peroxidation. Studies suggest that the hydrophobicity of peptides is important for the lipid peroxidation inhibitory activity [18]. However, the hydrophobic groups are easily wrapped in macromolecular peptides. Hence, low relative molecular mass fractions may be more easily dissolved in the lipid system to display lipid peroxidation inhibition activity. Similar observations have been reported in *Dosidicus gigas* skin gelatin peptides [32] and in *Dosidicus gigas* muscle peptides [33].

## 4. Conclusions

To explore the feasibility of the application of *Cyprinus carpio haematopterus* scale gelatin in functional food, a simulated digestion process was performed on *Cyprinus carpio haematopterus* scale gelatin. After the simulated digestion process, the degree of hydrolysis of *Cyprinus carpio haematopterus* scale gelatin reached about 16.9%. The result showed that *Cyprinus carpio haematopterus* scale gelatin had certain nutritional values because of its high digestibility. The *Cyprinus carpio haematopterus* scale gelatin digested products exhibited antioxidant activity in five different test models in vitro. The fraction of 1–3 kDa and the fraction of <1 kDa showed good ability to scavenge hydroxyl and DPPH radicals. The *Cyprinus carpio haematopterus* scale gelatin digested products can also chelate metal cations to reduce the yield of radicals such as hydroxyl radicals produced in iron-catalyzed conversion of H_2_O_2_. The antioxidant activity of *Cyprinus carpio haematopterus* scale gelatin digested products indicated that the gelatin from *Cyprinus carpio haematopterus* scale can be used as functional food raw material. In addition, it is more convenient to use gelatin than gelatin peptide as raw material. In particular, peptides may be hydrolyzed after entering the gastrointestinal tract, leading to changes in their activity. Therefore, simulated digested products can more accurately reflect the function of *Cyprinus carpio haematopterus* scale gelatin. To illustrate the property–structure relationship of *Cyprinus carpio haematopterus* scale gelatin digested products, the highly active peptide molecules need to be separated and purified.

## Figures and Tables

**Table 1:** Basic components of *Cyprinus carpio haematopterus* scale.

Component	Moisture	Mineral	Protein	Collagen
Content (%)	16.29 ± 0.69%	24.67 ± 0.08%	58.38 ± 1.07%	13.43 ± 0.46%

**Table 2:** The content of protein and proportion of free amino groups in three fractions of scale gelatin hydrolysates during a simulated gastrointestinal digestion.

	JCP1 (>3 kDa)	JCP2 (1–3 kDa)	JCP3 (<1 kDa)
Protein content (%)	46.2 ± 3.1	29.3 ± 1.9	23.5 ± 2.6
Free amino groups (%)	16.5 ± 2.5	19.3 ± 2.8	61.8 ± 3.4

## Abbreviations

GI	gastro-intestinal
DPPH	1,1-diphenyl-2-picrylhydrazyl
DH	hydrolysis degree
JCP1	>3 kDa fraction isolated *Cyprinus carpio haematopterus* scale gelatin digested products
JCP2	1–3 kDa fraction isolated *Cyprinus carpio haematopterus* scale gelatin digested products
JCP3	<1 kDa fraction isolated *Cyprinus carpio haematopterus* scale gelatin digested products
